# Development of a multimodal model combining radiomics and deep learning to predict malignant cerebral edema after endovascular thrombectomy

**DOI:** 10.3389/fneur.2025.1716443

**Published:** 2025-11-27

**Authors:** Jiayi Hong, Jiahong Fu, Feifan Liu, Yuhan Chen, Yujie Shen, Yan Li, Sheng Hu, Jingjing Fu

**Affiliations:** 1 Department of Neurology, The Fourth Affiliated Hospital of School of Medicine, and International School of Medicine, International Institutes of Medicine, Zhejiang University, Yiwu, Zhejiang, China; 2 Department of Radiology, The Fourth Affiliated Hospital of School of Medicine, and International School of Medicine, International Institutes of Medicine, Zhejiang University, Yiwu, Zhejiang, China

**Keywords:** thrombectomy, acute ischemic stroke, deep learning, radiomics, multimodal fusion

## Abstract

**Background:**

Malignant cerebral edema (MCE) represents a severe complication after endovascular thrombectomy (EVT) in treating acute ischemic stroke. This study aimed to develop and validate a multimodal predictive model integrating clinical data, radiomics features, and deep learning (DL)-derived features to improve the accuracy of MCE risk prediction following EVT.

**Methods:**

A total of 290 patients were included, comprising 189 in the training, 47 in the validation, and 54 in the internal test cohorts. A fusion model was developed by integrating clinical variables, radiomics, and DL features. Separate models based on clinical data, radiomics, and DL features were also constructed for comparison. Model training and evaluation were performed on training, validation, and test cohorts. The predictive performance of the combined model was compared with the ACORNS grading scale using an area under the curve (AUC) analysis to assess clinical effectiveness.

**Results:**

The combined model exhibited the best predictive performance. Analysis of the receiver operating characteristic curve revealed an AUC of 0.927 [95% confidence interval (CI): 0.849–1.000] for predicting MCE in the validation group and an AUC of 0.924 (95% CI: 0.846–1.000) in the test group. Additionally, the fusion model consistently demonstrated higher net benefits across all threshold probabilities than the ACORNS grading scale.

**Conclusions:**

This study integrated clinical data, radiomics, and DL features to develop a multimodal predictive model with a strong discriminative ability to predict MCE after EVT.

## 1 Introduction

Endovascular thrombectomy (EVT) has proven to be an effective intervention for acute ischemic stroke (AIS) resulting from large vessel occlusion in the anterior circulation (1). However, this treatment carries significant risks, particularly the development of malignant cerebral edema (MCE), which can undermine the clinical benefits of the procedure and may lead to death (2). Current evidence indicates that early decompressive craniectomy considerably reduces mortality rates (2–4). Consequently, developing reliable methods for the early detection and precise prediction of post-EVT MCE is essential for optimizing treatment strategies and improving clinical outcomes (5).

Existing predictive models for MCE following EVT primarily include the ACORNS grading scale (6) and clinical nomograms (7–9), all of which have demonstrated favorable outcomes. It should be noted that in a hospital-based clinical series, decreased level of consciousness, nausea or vomiting, and heavy smoking were identified as the main predictive clinical factors associated with malignant middle cerebral artery infarction (10). However, many existing models often depend on parameters susceptible to subjective interpretation and technical variability, undermining their predictive accuracy and clinical reliability. Recent studies have emphasized the significance of hyperattenuated imaging markers (HIM) as objective imaging biomarkers in predicting MCE after EVT (11, 12). HIM refers to areas of abnormal hyperdensity observed on noncontrast head computed tomography (NCCT) immediately post-EVT, detectable in up to 87% of patients' CT images (13). Emerging evidence suggests this radiological phenomenon may indicate blood-brain barrier disruption following reperfusion injury (14). Previous studies have confirmed that the extent and severity of HIM are effective predictors of MCE after EVT in patients with a stroke (11, 12).

Artificial intelligence (AI) technology has recently made significant advancements in predicting MCE following EVT in patients with AIS (14–16). A Previous study (17) reported strong predictive performance (AUC = 0.879 in the test set) using a combined CT-based radiomics and clinical model. Notably, multimodal fusion strategies integrating clinical data, radiomics, and deep learning (DL) techniques have demonstrated considerable success in oncology. Studies reported that multimodal models demonstrate significantly higher accuracy than single-modality models in differentiating ovarian tumors from thymomas (18, 19). Therefore, we hypothesized that multimodal fusion strategies may enhance the precision of predictive systems for MCE after EVT.

This study developed a multimodal model that integrates clinical data, radiomics features, and DL techniques to improve the accuracy of MCE risk prediction following EVT.

## 2 Materials and methods

### 2.1 Ethical approval of the study protocol

The study received approval from the Ethics Committee of the Fourth Affiliated Hospital of the School of Medicine and International School of Medicine, International Institutes of Medicine, Zhejiang University (Approval Number: K2024139). The committee exempted informed consent. All clinical studies complied with the principles of the Declaration of Helsinki.

### 2.2 Patient cohort

A dataset of post-EVT cases for large-vessel occlusive stroke in the anterior circulation was used, collected from the Fourth Affiliated Hospital of the School of Medicine and the International School of Medicine, International Institutes of Medicine, Zhejiang University, between September 2016 and March 2023. The model was validated in a test cohort from April 2023 to January 2024 at the same hospital to evaluate clinical applicability and performance with real-world data.

**Figure 1:**
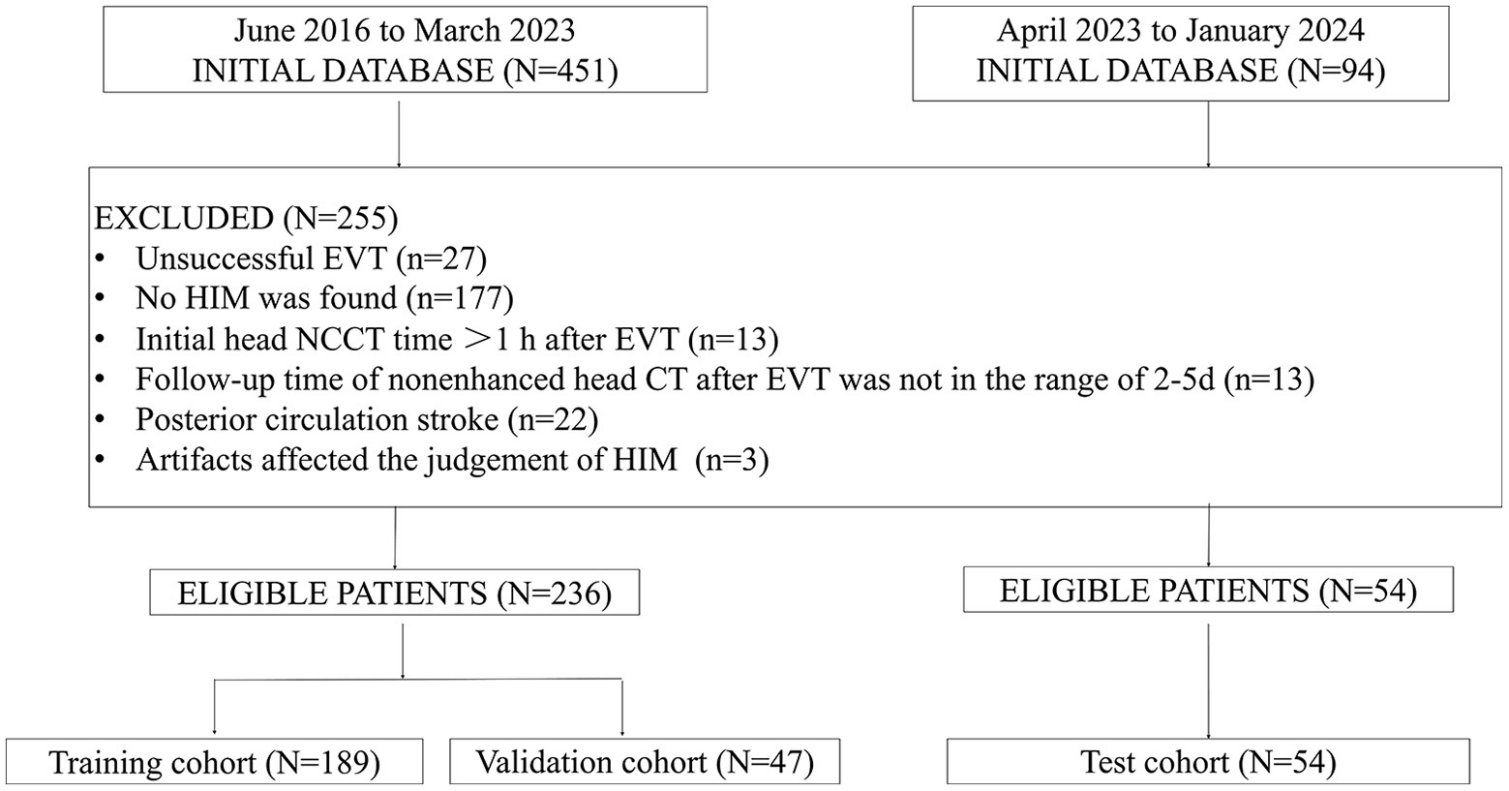
Flowchart of patient enrollment. EVT, endovascular thrombectomy; HIM, hyperattenuated imaging marker; NCCT, non-contrast computed tomography; CT, computed tomography.

We enrolled patients who (1) had a diagnosis of AIS confirmed by diffusion-weighted imaging or CT; (2) received EVT as treatment; (3) underwent a head NCCT within 1 h following EVT; (4) demonstrated HIM, characterized by increased attenuation in the brain parenchyma and/or subarachnoid space. The exclusion criteria were as follows: (1) Follow-up NCCT performed outside the 2–5 day window after EVT, (2) artifacts, such as metal or motion artifacts, that interfered with the assessment of HIM or MCE, and (3) patients with posterior circulation stroke. Figure 1 presents a flowchart detailing participant inclusion in this study.

### 2.3 Clinical data

Data on general clinical features, laboratory examinations, clinical presentations were analyzed. Patient charts, procedure notes, and follow-up records were reviewed to extract baseline and preprocedural factors, including age, sex, history of alcohol consumption or smoking, hypertension, hyperlipidemia, diabetes mellitus, atrial fibrillation, coronary artery disease, and baseline National Institute of Health Stroke Scale (NIHSS) score. Additionally, modified thrombolysis in cerebral infarction scores and various pre-interventional parameters, including intravenous thrombolysis therapy, were collected. Interventional parameters were also recorded, such as the percentage of stent implantation, stent pass number, and type of stent used.

### 2.4 Data preprocessing

A total of 290 patients were enrolled in this study. Of these, 236 patients were randomly assigned to the training cohort (189 cases) and the validation cohort (47 cases). An additional 54 patients were included in the internal test cohort.

Images of the NCCT were obtained using a 64-row spiral CT scanner (SOMATOM Definition AS, Siemens Healthineers, Erlangen, Germany) and a 62-row spiral scanner (Optima CT620, GE HealthCare, Chicago, IL, USA). The scanning parameters included axial mode, tube voltage of 120 kV, tube current of 250–300 mAs, scanning range from the skull base to the cranial roof, section thickness of 5 mm, and reconstruction using the standard algorithm. Imaging data were evaluated independently by two trained junior neuroradiologists blinded to clinical outcome and demographic data. All imaging data were evaluated with subsequent consensus reading of one senior neuroradiologist and analyzed over a consecutive period of 12 weeks.

### 2.5 Image analysis

Neuroradiologists independently assessed the Alberta Stroke Program Early CT Score (ASPECTS) on baseline NCCT images. HIM were evaluated on NCCT images within 1 h after EVT, characterized by increased attenuation in the brain parenchyma and/or subarachnoid space. The neuroradiologists analyzed the maximum Hounsfield unit (HUmax) and the volume of the HIM within the region of interest (ROI). The HUmax was defined as the highest HU value within a specified ROI, measuring 3 × 3 pixels.

### 2.6 Outcome measures

A review of NCCT images obtained after EVT was conducted, with the development of MCE was defined as the occurrence of midline brain shift, specifically a displacement of the septum pellucidum of ≥5 mm, within the first 5 days after admission. Two neuroradiologists independently evaluated all datasets without knowledge of patient outcomes. Any discrepancies were resolved by consensus.

### 2.7 Feature extraction and selection

Radiomic features were extracted from HIM in NCCT using the Pyradiomics in-house feature analysis program (http://pyradiomics.readthedocs.io). A total of 1,834 radiomic features were extracted, including shape features, first-order features, gray co-occurrence matrix features, gray region size matrix features, gray stroke matrix features, neighborhood gray difference matrix features, gray correlation matrix features, and wavelet features.

DL features based on the HIM region of interest (ROI) were extracted from five commonly used DL models for modeling, including ResNet152, Inception v3, ResNet50, Visual Geometry Group (VGG) 16, and VGG19. We compared and selected the best DL model and used it as a pre-trained model. Data augmentation techniques, such as RandomResizedCrop and RandomHorizontalFlip, were applied to enhance the model's generalization ability. DL features were extracted from CT images and selected after dimensionality reduction via principal component analysis (PCA).

### 2.8 Development and validation of the model

Ultimately, the multi-omics features consisted of clinical data, radiomics, and DL features. These features were standardized to have a mean of 0 and a variance of 1. Feature selection was performed using analysis of variance (ANOVA), Pearson's correlation coefficient, PCA, and Least Absolute Shrinkage and Selection Operator (LASSO) to improve the model's generalization ability and minimize overfitting. For clinical, radiomics, and DL models, ANOVA, Pearson's correlation coefficient, and LASSO were sequentially applied for feature selection. PCA, ANOVA, Pearson's correlation coefficient, and LASSO were sequentially utilized for the combined model. Additionally, individual models based on clinical data, radiomics, and DL features were also constructed for comparison. MCE risk prediction was performed using seven different classifiers. The optimal classifier was selected based on predictive performance in validation and test cohorts. The performance of these models was evaluated using accuracy, sensitivity, specificity, receiver operating characteristic (ROC) curves, area under the curve (AUC), and decision curve analysis (DCA). Calibration curves were plotted to assess the agreement between the predicted and actual classifications, thereby evaluating the calibration efficiency of each model.

### 2.9 Comparison of the model with the ACORNS grading scale

**Figure 2:**
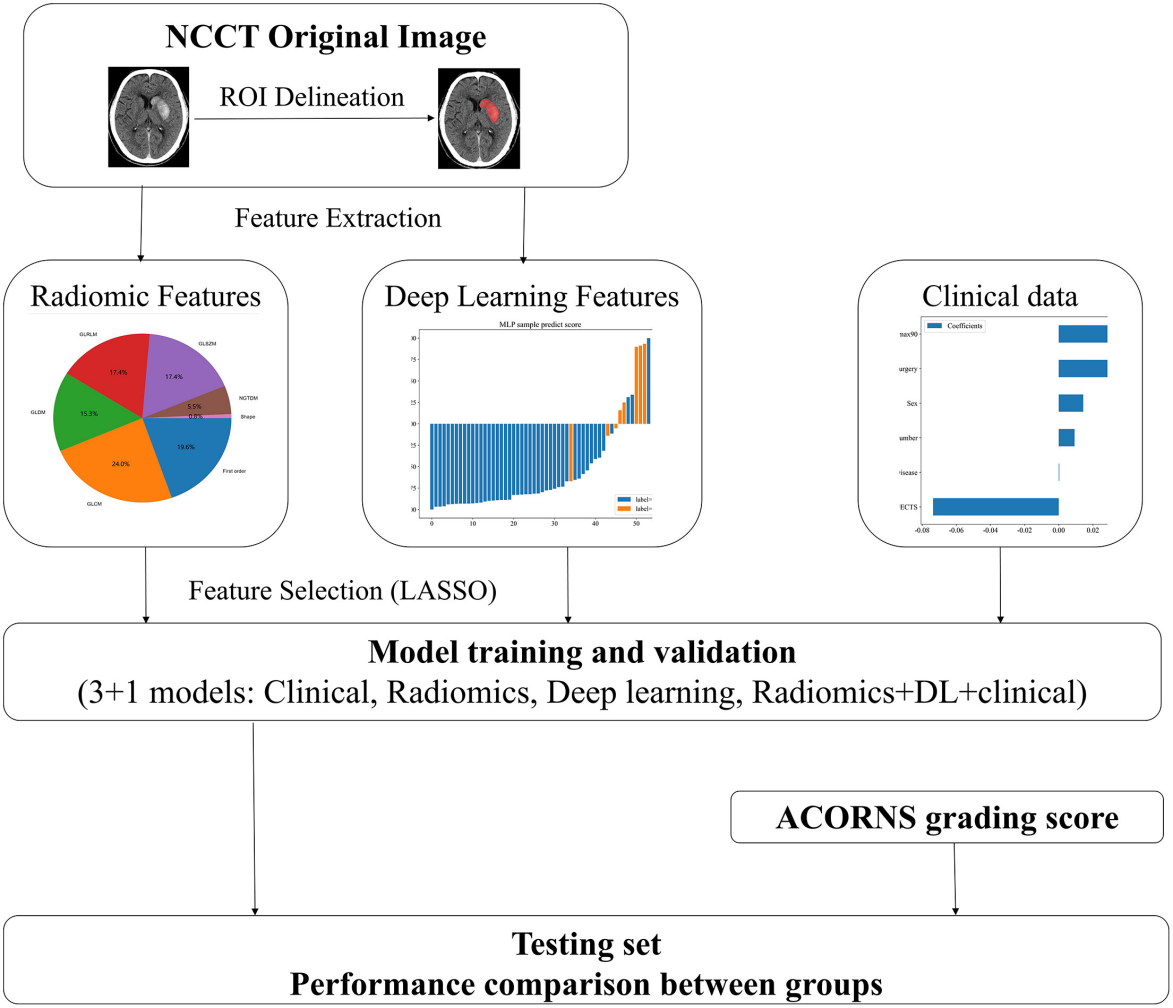
The overall pipeline of this study.

Huang et al. (6) developed the ACORNS grading score based on clinical predictors, including hypertension history, baseline NIHSS score, ASPECT score, glycemia, collateral circulation status, occlusion location, thrombolysis prior to thrombectomy, and reperfusion status. This score demonstrated strong predictive performance. The same variables were collected to compare with the AI model, and an external validation of the ACORNS grading score was conducted. The workflow and global analysis pipeline for the classification model are presented in Figure 2.

### 2.10 Statistical analysis

Statistical analysis was conducted using the Statistical Package for the Social Sciences software (version 26.0, IBM Corp., Armonk, NY, USA). The normality of the evaluated variables was assessed using the Kolmogorov–Smirnov and Shapiro–Wilk tests. Continuous variables are presented as mean ± standard deviation, while categorical variables are reported as frequency counts and percentages. Categorical variables were compared using the chi-square or Fisher's exact test, and continuous variables were compared using the Mann–Whitney *U*-test or independent *t*-test. Feature extraction, screening, and model construction were performed using Python software (version 3.11.1, Python Software Foundation, Wilmington, DE, USA). Model performance was evaluated using AUC with 95% confidence interval (CI), accuracy, sensitivity, and specificity. A *p*-value of < 0.05 was considered statistically significant for all tests.

## 3 Results

### 3.1 Patient baseline characteristics

This study included 290 patients, with 189 in the training, 47 in the validation, and 54 in the test cohorts. Table 1 summarizes the baseline demographic, clinical, and imaging characteristics of the patients. Significant differences were observed between the training, validation, and test cohorts regarding thrombolysis (*p* = 0.006) and aspiration catheter (*p* = 0.000). MCE occurred in 58 of the 290 patients. The overall in-hospital mortality was 6.9% (20/290). The hospital mortality rate was significantly higher in the MCE subgroup (24.1%, 14/58) than in the non-MCE subgroup (2.6%, 6/232; Fisher's exact test, *p* < 0.001). The presence of MCE was associated with an 11.83-fold increase in the odds of death (OR= 11.83, 95% CI: 4.01–39.71).

**Table 1:** Characteristics of the training, validation and test sets.

**Feature name**	**Training**	**Validation**	**Test**	***p*-value**
Age (years), mean ± SD	66.20 ± 15.00	70.62 ± 11.14	66.17 ± 18.04	0.185
Males, *n* (%)	119 (62.96)	29 (61.70)	37 (68.52)	0.718
Atrial fibrillation, *n* (%)	75 (39.68)	23 (48.94)	23 (42.59)	0.513
Hypertension, *n* (%)	111 (58.73)	28 (59.57)	31 (57.41)	0.975
Hyperlipidemia, *n* (%)	15 (7.94)	4 (8.51)	9 (16.67)	0.154
Diabetes mellitus, *n* (%)	28 (14.81)	7 (14.89)	9 (16.67)	0.945
Coronary artery disease, *n* (%)	21 (11.11)	8 (17.02)	3 (5.56)	0.187
Drinking, *n* (%)	34 (18.00)	13 (27.66)	9 (16.67)	0.281
Smoking, *n* (%)	43 (22.75)	8 (17.02)	12 (22.22)	0.694
Thrombolysis, *n* (%)	70 (37.04)	21 (44.68)	9 (16.67)	0.006^*^
Baseline NIHSS, median (Q1, Q3)	15 (12–19)	18 (15–21)	15 (10–20)	0.084
ASPECTS, median (Q1, Q3)	9 (8–10)	9 (8–10)	9 (9–10)	0.280
HUmax ≥90, *n* (%)	54 (28.57)	15 (31.91)	16 (29.63)	0.903
mTICI beyond 2b, *n* (%)	173 (91.53)	42 (89.36)	51 (94.44)	0.646
Stent type, *n* (%)				0.526
Solitaire	111 (58.73)	28 (59.57)	32 (59.26)	
Trevo	24 (12.70)	11 (23.40)	0 (0.00)	
Solitaire+trevo	32 (16.93)	3 (6.38)	16 (29.63)	
Others	22 (11.64)	5 (10.64)	6 (11.11)	
Pass number, median (Q1, Q3)	2 (1–3)	2 (1–3)	2 (0–2)	0.364
Aspiration catheter, *n* (%)	71 (37.57)	9 (19.15)	45 (83.33)	0.000^*^
Extravasation during surgery, *n* (%)	10 (5.29)	1 (2.13)	3 (5.56)	0.641
Stent implantation, *n* (%)	36 (19.05)	10 (21.28)	7 (12.96)	0.505

SD, standard deviation; ASPECTS, alberta stroke program early computed tomography score; Q1, first quartile; Q3, third quartile; NIHSS, National Institutes of Health Stroke Scale; HU, Hounsfield; mTICI, modified thrombolysis in cerebral infarction. ^*^P < 0.05.

### 3.2 Performance of different models

The fusion model was conducted using the ResNet152 model and the MLP classifier, which demonstrated the highest performance across the training, validation, and test cohorts. In the training cohort, the fusion model achieved an AUC of 0.978 (95% CI: 0.961–0.995), with a sensitivity of 0.950 and a specificity of 0.872. In the validation cohort, the fusion model achieved an AUC of 0.927 (95% CI: 0.849–1.000), with a sensitivity of 0.700 and a specificity of 0.919. In the test cohort, the fusion model achieved an AUC of 0.924 (95% CI: 0.846–1.000), with a sensitivity of 0.750 and a specificity of 0.913. Predictive performances of each classifier in test cohort are provided in Table 2.

**Table 2:** Predictive performance of each machine learning model in test cohort.

**Model name**	**AUC (95% CI)**	Sensitivity^*^	Specificity^*^	**Accuracy**	**PPV**	**NPV**
NaiveBayes	0.887 (0.802–0.973)	0.875	0.826	0.833	0.467	0.974
SVM	0.902 (0.809–0.996)	0.75	0.826	0.815	0.429	0.95
RandomForest	0.908 (0.814–1.000)	0.75	0.783	0.778	0.375	0.947
ExtraTrees	0.864 (0.741–0.988)	0.625	0.891	0.852	0.5	0.932
XGBoost	0.856 (0.698–1.000)	0.625	0.913	0.87	0.556	0.933
LightGBM	0.838 (0.636–1.000)	0.5	0.978	0.907	0.8	0.918
MLP	0.924 (0.846–1.000)	0.75	0.913	0.889	0.6	0.955

^*^Balanced sensitivity and specificity at the cutoff yielding the largest Youden index value.

AUC, area under the receiver operating characteristic curve; CI, confidence interval; PPV, positive predictive value; NPV, negative predictive value.

**Figure 3:**
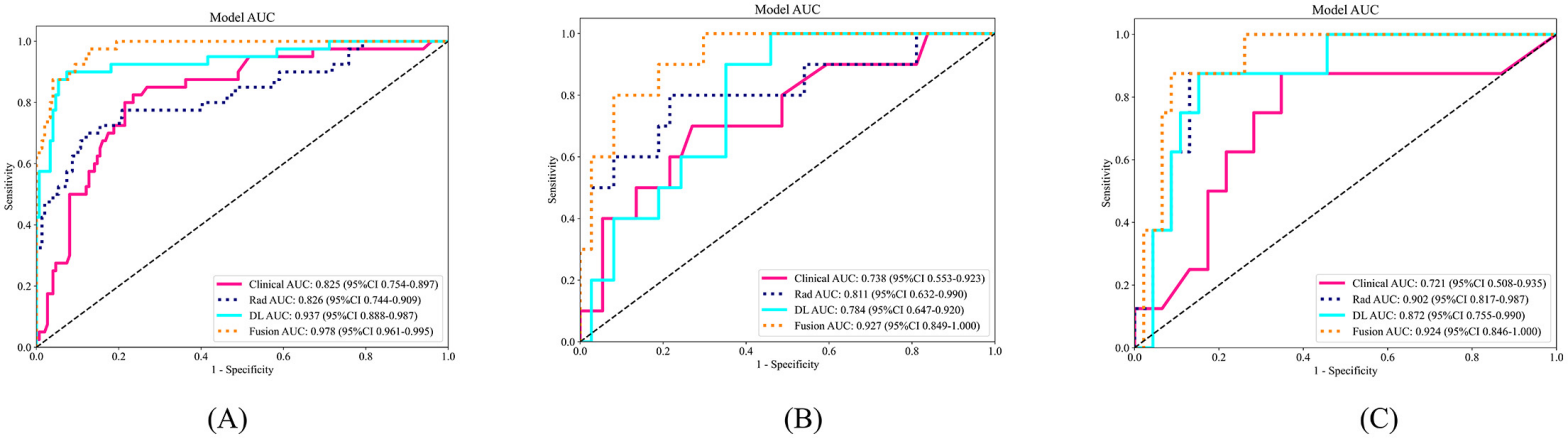
ROC curves of the clinical model, radiomics model, deep learning model and fusion model in the training cohort **(A)**, validation cohort **(B)** and test cohort **(C)**. ROC, receiver operating characteristic.

**Figure 4:**
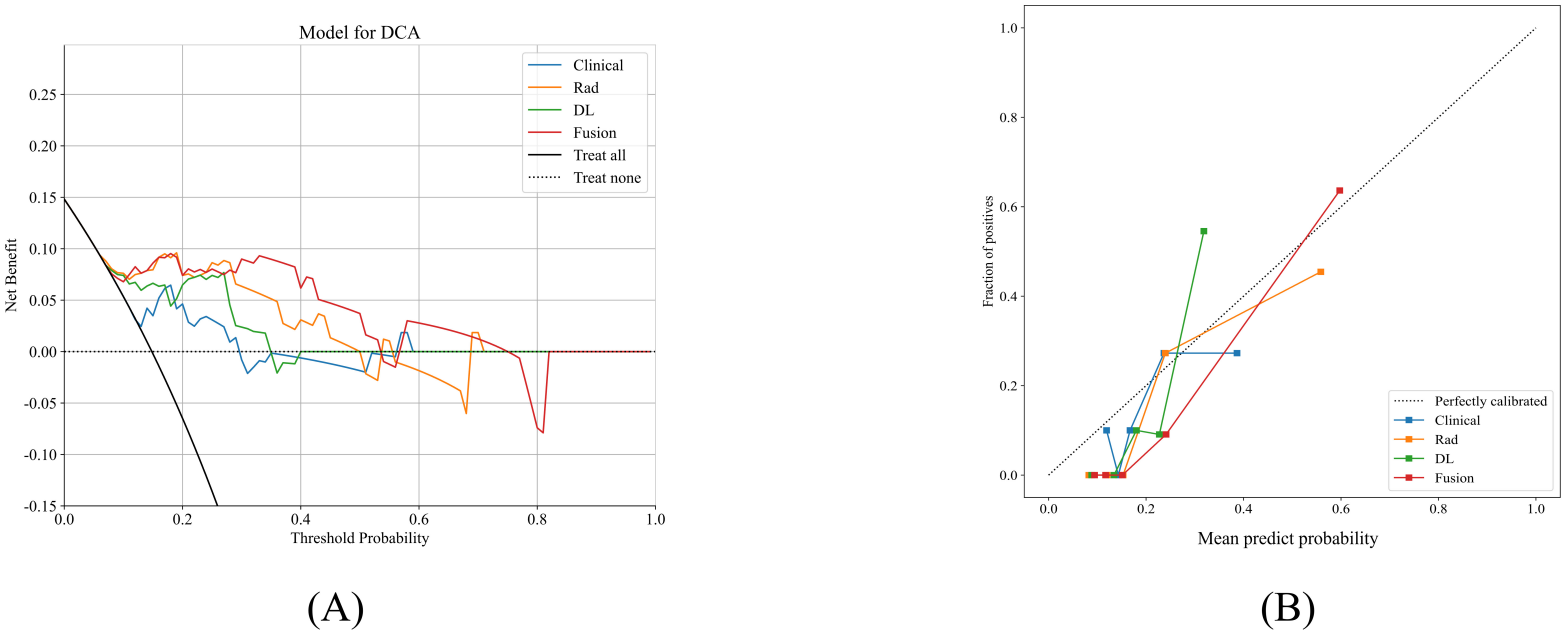
The DCA curves and calibration curves of all the models in test cohort. **(A)** DCA curves of different models in the test cohort. The *y*-axis represents the net benefit, and the *x*-axis represents the threshold probability. **(B)** Calibration curves of different models in the test cohort; the closer the solid line was to the dotted line, the better of the predictive power of the models. DCA, decision curve analysis.

The predictive performance was also compared across clinical, radiomics, and DL models in the training, validation, and test cohorts. In the test cohort, AUC values were as follows: 0.924 for the fusion model (incorporating radiomics, DL, and clinical data), 0.721 for the clinic model, 0.902 for the radiomics model, and 0.872 for the DL model. Detailed model parameters in test cohort are provided in Table 3. Figure 3 presents a comparison of ROC curves and AUC values of the different models in the training, validation, and test cohorts. Figure 4 illustrates DCA and calibration curves for various models in the test cohort. The calibration curves revealed strong agreement between the model predictions and the actual MCE classifications in the test cohort, with the fusion model demonstrating the best fit. The DCA further highlighted that all models notably improved patient outcome predictions compared to scenarios without any prediction model. The fusion model provided the most substantial clinical benefit for automatic MCE classification.

**Table 3:** Predictive performance of each model in test cohort.

**Model name**	**AUC (95% CI)**	Sensitivity^*^	Specificity^*^	**Accuracy**	**PPV**	**NPV**
Clinical model	0.721 (0.508–0.935)	0.750	0.652	0.667	0.273	0.937
Radiomics model	0.902 (0.817–0.987)	0.750	0.870	0.852	0.500	0.952
Deep learning model	0.872 (0.755–0.990)	0.750	0.848	0.833	0.462	0.951
Fusion model	0.924 (0.846–1.000)	0.750	0.913	0.889	0.600	0.955

^*^Balanced sensitivity and specificity at the cutoff yielding the largest Youden index value.

AUC, area under the receiver operating characteristic curve; CI, confidence interval; PPV, positive predictive value; NPV, negative predictive value.

### 3.3 Comparison of the fusion model with the ACORNS grading scale

**Figure 5:**
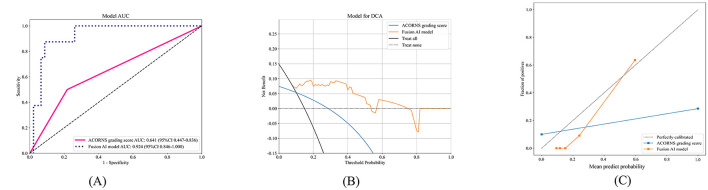
The ROC curves **(A)**, DCA curves **(B)** and Calibration curves **(C)** of the ACORNS grading score model and our fusion model in test cohort. ROC, receiver operating characteristic.

The ACORNS grading scale's clinical utility and discriminatory performance were evaluated and compared with the fusion model using DCA and ROC curves. In the test cohort, the AUC for the ACORNS grading scale was 0.641 (95% CI: 0.447–0.836). Additionally, the clinical effectiveness of the ACORNS grading scale and the combined model were compared using a DCA curve, revealing that the fusion model consistently provided higher net benefits across all threshold probabilities than the ACORNS grading scale. Figure 5 depicts a comparison of AUC values, DCA curves, and calibration curves for the two models in the test cohort.

## 4 Discussion

This study developed a fusion AI model based on the HIM framework, integrating clinical data, radiomic signatures, and DL-derived features using an ensemble machine learning classifier. Internal validation demonstrated strong discriminative performance, achieving AUC values of 0.978 in the training cohorts and 0.927 in the validation cohort. A subsequent 10-month evaluation confirmed improved predictive capability (AUC = 0.924), surpassing the conventional ACORNS grading scale (AUC = 0.641) in prognostic accuracy and clinical relevance.

In this study, comprehensive screening identified ASPECTS score and HUmax ≥ 90 as the two clinical features most strongly associated with MCE, aligning with previous research findings (20, 21). The ASPECTS score quantifies early ischemic changes on CT imaging and serves as an indicator of stroke severity; lower scores indicate a higher risk of MCE (20). Additionally, HUmax ≥ 90 reflects significant blood-brain barrier disruption (21), which is associated with a higher risk of MCE.

Radiomics and DL enable quantitative extraction of subvisual pathological information through advanced image feature analysis compared to subjective radiological assessments (19). Extensive evidence supports the prognostic value of radiomics in predicting cerebral edema following EVT, with Wen et al.'s (17) clinical-radiomics model achieving an AUC of 0.879 for MCE detection. A subsequently developed HIM-incorporated nomogram (14) demonstrated enhanced predictive performance. However, the limited robustness of radiomic features to spatial transformation restricts clinical generalizability, while DL architectures provide automated global feature representation, highlighting methodological complementarity. Moreover, DL features may exhibit sensitivity to global translation, rotation, and scaling of images, whereas radiomics features remain unaffected by such variations (22). Transfer learning has emerged as an effective strategy to improve cross-domain feature transferability and address the challenge of limited medical imaging data scarcity (23). A previous study (24) employed ResNeXt-101 to analyze HIM on NCCT following EVT, yielding promising results in predicting MCE. These findings demonstrated that HIM provides a robust dataset for AI applications and validated the technical feasibility of predicting post-EVT MCE by integrating radiomic features with DL architectures. This evidence establishes a critical foundation for developing multimodal predictive systems in neurocritical care.

Radiomics, DL features, and clinical data capture distinct pathophysiological characteristics of MCE, leading to the hypothesis that multimodal fusion strategies can enhance predictive accuracy for post-EVT MCE. Successful cross-domain applications support this theoretical framework; Jan et al. (19) developed a CT-based paradigm that integrated radiomics and DL features to differentiate benign from malignant ovarian tumors, while Wang et al. (22) combined DL, radiomics, and clinical data to predict lymph node metastasis in laryngeal squamous cell carcinoma. All these studies achieved favorable results. Despite these advancements, the synergistic integration of radiomics and DL for post-EVT MCE prediction remains unexplored. This study constructed independent models based on clinical data, radiomics, and DL features. The multimodal fusion model demonstrated superior performance, achieving stable accuracy rates of 0.927 in the validation and 0.924 in the test cohorts, significantly surpassing unimodal approaches. This enhanced predictive capability supports the hypothesis that multimodal integration of clinical, radiomic, and DL biomarkers provides complementary insights for MCE, resulting in a more effective synergistic effect for predicting post-EVT MCE.

The ACORNS grading scale (6), which incorporates eight features, including the NIHSS score and the ASPECTS, predicts the risk of MCE in patients with stroke and large vessel occlusion following EVT. It demonstrated robust discriminative performance, with AUC values of 0.850, 0.874, and 0.785 in the training, internal validation, and external validation cohorts. This study further evaluated the predictive performance of the ACORNS grading scale through an external validation cohort (AUC = 0.641), reaffirming its predictive capability for MCE. However, the proposed model integrates clinical data, radiomic signatures, and DL features. These features can quantify disease characteristics and detect subclinical alterations undetectable by conventional clinical assessments, resulting in superior predictive performance.

An interesting yet contrasting finding of our study was the identification of male gender as an independent risk factor for MCE, which diverges from some previous reports, including a meta-analysis suggesting female gender as an independent risk factor (25). This discrepancy may be attributable to several factors specific to our study. Firstly, the high prevalence of risk factors among male stroke patients in China, such as smoking, alcohol consumption (26), may contribute to a greater burden of pre-existing cerebrovascular disease, potentially increasing susceptibility to MCE. Secondly, potential biases in our study must be considered. The predominance of males in our single-center cohort (63.79%, 185/290), which is susceptible to referral bias as a comprehensive stroke center, may reflect a population with more severe strokes, influencing the observed association. Furthermore, our cohort was exclusively composed of patients exhibiting HIM on post-EVT CT scans, which may introduce selection bias, limiting the generalizability of our gender-specific finding to the broader EVT population. Finally, despite multivariate adjustment, residual confounding by unmeasured variables, such as collateral status, genetic factors, cannot be ruled out. Therefore, the relationship between gender and MCE risk warrants further investigation in larger, prospective, and multi-center studies.

This study has several limitations that should be acknowledged. First, our study cohort exclusively included patients who exhibited HIM on post-EVT NCCT. While this selection criterion was essential for model development targeting this specific imaging phenotype, it inevitably introduces a potential selection bias. Consequently, the applicability of our proposed model is currently restricted to this patient subgroup, and its predictive performance in the broader, unselected EVT population remains uncertain and warrants future investigation. Second, the single-center design with a modest sample size (*n* = 290) and the lack of external validation cohorts may limit the model's generalizability and increase the risk of overfitting. Third, an important technical limitation pertains to the CT data acquisition. The scans were acquired using scanners from different vendors (Siemens and GE) with heterogeneous acquisition and reconstruction parameters. As no harmonization techniques (e.g., ComBat) or intensity normalization procedures were applied, the inherent scanner-related variability could have influenced the stability of extracted radiomic features, potentially affecting the model's performance and robustness. Fourth, the ROI for the HIM was delineated manually, which was time-consuming and may introduce interobserver variability. Fifth, although the strategy of our feature reduction pipeline may improve internal predictive performance, it introduces complexity that raises concerns about reproducibility. Finally, the interpretability of the model is limited, as it is unclear which features contribute most to prediction.

Based on the findings and limitations identified in this study, several strategic directions for future research are recommended. First, external validation through large-scale, prospective, multicenter cohorts is essential to verify the model's generalizability and robustness. Second, the clinical workflow could be streamlined by developing deep learning-based algorithms for the fully automated segmentation of HIM along with using more advanced approaches such as habitat analysis to enable finer-grained examination of lesion characteristics. In parallel, the interpretability of the model must be improved using techniques, such as SHAP (SHapley Additive exPlanations) and Grad-CAM (Gradient-weighted Class Activation Mapping), to clarify the contribution of key features. Lastly, future work could focus on constructing dynamic prediction models that integrate serial imaging data, such as follow-up CT or MRI, to monitor edema progression in real time, which may facilitate preemptive clinical interventions.

In conclusion, this study introduces a novel and comprehensive approach that integrates clinical, radiomic, and DL models, offering a rapid and effective method to predict MCE after EVT. With its robust predictive capability, the fusion model has significant potential as a practical tool for clinicians.

## Data Availability

The datasets presented in this article are not readily available because all data are required for further research. Requests to access the datasets should be directed to Jingjing Fu, fujingjing1985@zju.edu.cn.
